# Can the imaging manifestations of melioidosis prognosticate the clinical outcome? A 6-year retrospective study

**DOI:** 10.1186/s13244-019-0708-8

**Published:** 2019-02-13

**Authors:** Hannah L. Khiangte, Leena Robinson Vimala, Balaji Veeraraghavan, Binesh Lal Yesudhason, Reka Karuppusami

**Affiliations:** 1 Department of Radiodiagnosis, Christian Medical College Vellore, Tamil Nadu, 632004 India; 2 Department of Microbiology, Christian Medical College Vellore, Tamil Nadu, 632004 India; 3 Department of Biostatistics, Christian Medical College Vellore, Tamil Nadu, 632004 India

**Keywords:** Melioidosis, Imaging, Prognosis, Risk factors

## Abstract

**Objective:**

Melioidosis being an important cause of community-acquired sepsis, caused by *Burkholderia pseudomallei* in the tropical and subtropical countries, is often underreported or misinterpreted on imaging investigations. We aim to describe the spectrum of imaging manifestations of melioidosis and to evaluate its role in prognosticating clinical outcome, and look for association of specific organ involvement with risk factors.

**Methods:**

From January 2011 to October 2017, retrospective analysis of imaging investigations of 189 consecutive patients with culture-proven melioidosis was performed. Clinical and demographic records were collected from the hospital medical records.

**Results:**

Out of 67% with a localised disease musculoskeletal involvement was most common, whereas the common organs involved in disseminated infections were the lungs, spleen, liver and genitourinary tract in descending order. Twenty percent suffered unfavourable outcome with a mortality rate of 8.5%. The lung involvement was associated with unfavourable outcome (OR 3.2 [95%CI 1.54–6.63] *p* = 0.002). The lymph node involvement (OR 0.22 [95% CI 0.05–0.95] *p* = 0.04) predicted a favourable outcome. Those with diabetes were at a higher risk of splenic (OR 3.05 [95% CI 1.62–5.77]; *p* = 0.001) and musculoskeletal involvement (OR 2.14 [95% CI 1.09–4.17] *p* = 0.03) of melioidosis.

**Conclusions:**

In this study, we have described the spectrum of imaging manifestation of melioidosis and evaluated its association with clinical outcome. Respiratory system involvement in melioidosis showed significant association with unfavourable outcome. Diabetes mellitus, a common risk factor for melioidosis, is more prone for infection of the spleen and musculoskeletal system. Thus awareness of imaging manifestations of melioidosis can complement microbiological diagnostic tests for accurate early diagnosis and management.

## Key points

- Disseminated form of melioidosis is common than localised disease
- Abscesses in the solid visceral organs may have associated chest or abdominal wall abscesses
- The presence of respiratory system involvement has stronger association for unfavourable outcome
- The spleen and musculoskeletal involvement of melioidosis is common in patients with diabetes mellitus.

## Introduction

Melioidosis is an important cause of community-acquired sepsis in the tropical and subtropical countries being well documented in Southeast Asia and northern Australia [1, 2]. Although sporadic case reports from India were noted by Cheng et al. [1] in 2005, most of the Indian subcontinent is recognised endemic for melioidosis since 2008 [3]. The disease is caused by a saprophytic gram-negative bacillus, *Burkholderia pseudomallei* (*B. pseudomallei*) [1, 4–6], freely living in soil and water. Human infection occur following percutaneous inoculation, inhalation of aerosolised bacteria or accidental ingestion; rarely person-to-person transmission, nosocomial transmission and laboratory-acquired infection can occur [1, 7]. Along with the expansion in human travel and movement, and climate change, there is a risk of imported infection worldwide and extension in the boundaries of endemic melioidosis [3, 8]. Clinical manifestations vary from localised cutaneous infections at sites of inoculation, chronic multisystem involvement mimicking tuberculosis to acute fulminant sepsis and death [9, 10]. Fatal cases have identified risk factors; the known risk factors are diabetes, excessive alcohol consumption, chronic renal and lung disease, thalassemia and other haematological conditions, immunocompromised state and occupational exposure to the pathogen [8, 11]. Mortality rates are 10 to 14% in Australia, 40 to 50% in Thailand and 30% in Malaysia [1, 5, 9, 12]. The reported mortality rate from the published literature in endemic countries varied from up to 58% in 2003 [13] and 9 to 25% in recent years [14, 15].

The standard of diagnosis is by the culture of a clinical specimen: however, challenges in laboratory diagnosis are lack of resources and expertise, misidentifying or underreporting of the bacilli with methods used routinely in clinical laboratories [16, 17]. Imaging is important in the evaluation of multisystem involvement of melioidosis; organ involvement manifests most commonly as abscesses [18]. Certain characteristics of abscesses caused by melioidosis or combinations of organ involvement may contribute to diagnosis in an appropriate clinical scenario [19, 20]. Though plain radiograph or ultrasonogram is often the first line of imaging, it is surpassed by cross-sectional studies in its usefulness to describe the extent and assess the complications of infection. There are studies that have analysed clinical and lab parameters indicating mortality [21, 22]; however, there are very few research in the usefulness of imaging in predicting the clinical outcome. In this study, we aim to describe the imaging manifestations of melioidosis and to analyse the organ involvement which could predict unfavourable outcome, and evaluate the association between the organ imaging manifestation and known risk factors/predisposing conditions.

## Methodology

### Data collection and study population

This study was performed in a tertiary referral centre in South India, in a cohort of patients in whom melioidosis was proven by blood, exudates, tissue, or urine culture in the period between January 2011 and October 2017, after the approval from the institutional review board. Diagnostic samples were obtained through blood culture of venous samples, aspiration of pus from abscesses, pleural fluid aspiration, urine culture, sputum culture, CSF culture and culture of catheter tips. Informed patient’s consent was waived as this was a retrospective study analysing existing records. Imaging studies were reviewed in the picture archiving and communicating system in the diagnostic radiology department. The routine diagnostic protocol in our institution is as follows: Abdominal symptoms are evaluated using ultrasonography, followed by computed tomography to detail the extent and severity of the disease. Chest symptoms are initially evaluated with chest radiograph, and if required, a computed tomographic scan of the thorax is performed. For patients with neurological symptoms, computed tomography scan or magnetic resonance imaging is performed. Depending on the requirement for joint and bone disease, plain radiograph, ultrasonography or cross-sectional studies are performed. Imaging findings in involved organs were noted. Demographic data, clinical characteristics, the presence of risk factors and clinical outcome of the disease were noted from patients’ laboratory records and the hospital information system.

### Definitions of variables

Localised melioidosis is defined as involvement of one organ system on imaging. For localised disease, culture positivity is defined as when the sample derived from the primary site or organ of involvement or blood culture yielded Burkholderia pseudomallei. Disseminated disease is defined as disease affecting more than one organ system on imaging [23]. In disseminated disease, culture positivity is defined as when the sample derived from organ/site of severe involvement or blood culture yielded Burkholderia pseudomallei. Septicaemia is defined as bacteraemia with hypotension not responsive to fluid replacement along with manifestation of one end-organ dysfunction [24].

Acute melioidosis is when symptoms are present for less than a 2-month duration, and chronic melioidosis is when symptoms are present for more than 2 months [7]. Favourable outcome was defined as patients discharged in stable condition with no requirement for critical care during the course of the illness. Unfavourable outcome was defined as patients with illness requiring ICU admission for administration of inotropes, requirement of ventilation and/or death.

## Statistical analysis

Continuous variables are presented as mean (SD) and categorical variables are presented as frequency (percentage). Pearson’s chi-square test was used to find the association between organ involvement and outcome (favourable and unfavourable) and mortality. Univariate binary logistic regression analysis was performed to evaluate the association of specific organ involvement with unfavourable outcome and the association of specific organ involvement with risk factors or predisposing conditions like diabetes mellitus and tuberculosis. A *p* value of < 0.05 was considered statistically significant. The SAS package (SAS® Institute Inc., USA, version 9.2) was used for Statistical evaluation.

## Results

### Demography and clinical data

One hundred ninety-four patients (158 males) with melioidosis were identified from the microbiological laboratory database during the study period. One hundred eighty-nine patients underwent various imaging tests which were included in the study sample. The age group of the cohort ranges from 9 months to 72 years, mean age being 42.9 (SD 16.3) years. The common risk factors amongst the study subjects were diabetes mellitus (65%), alcohol abuse (14%), haematological conditions like sickle cell anaemia (4%), chronic kidney disease (3%), heart disease (3%) and direct trauma to the skin (3%). Forty-six percent of the study cohort had an acute presentation. All the 189 cases included in the study were culture-proven melioidosis. Lab diagnosis of *Burkholderia pseudomallei* was made on pus culture in 95 patients and on blood culture in 79 patients. Culture from other body fluid samples grew *Burkholderia* in 42 patients (sputum 11, urine 11, pleural fluid 15, catheter tip 2, CSF3). In 14 patients, *Burkholderia pseudomallei* has been isolated from two different culture samples. Invasive methods like direct aspiration of pus, image-guided aspiration of pus and other body fluids, bronchoscopic guided aspiration and lavage, transbronchial lung biopsy and lumbar puncture for CSF analysis were performed for obtaining the diagnostic aspirate, according to the site of involvement. Venous samples were obtained from the cubital vein. Out of the 95 lab-positive pus culture during the study period, image guidance was used for the aspiration of pus from solid visceral organ abscesses in 35 patients.

### Imaging manifestations

Sixty-seven percent (126 patients) had localised disease and 33% (63 patients) had disseminated disease. The most common localised infection involved the musculoskeletal system (bones, joints, chest/abdominal wall). The common organs involved in disseminated infections were the lungs, spleen, liver and genitourinary tract in descending order. The various organ imaging manifestations encountered in this study are detailed below.

### Thorax

**Fig. 1:**
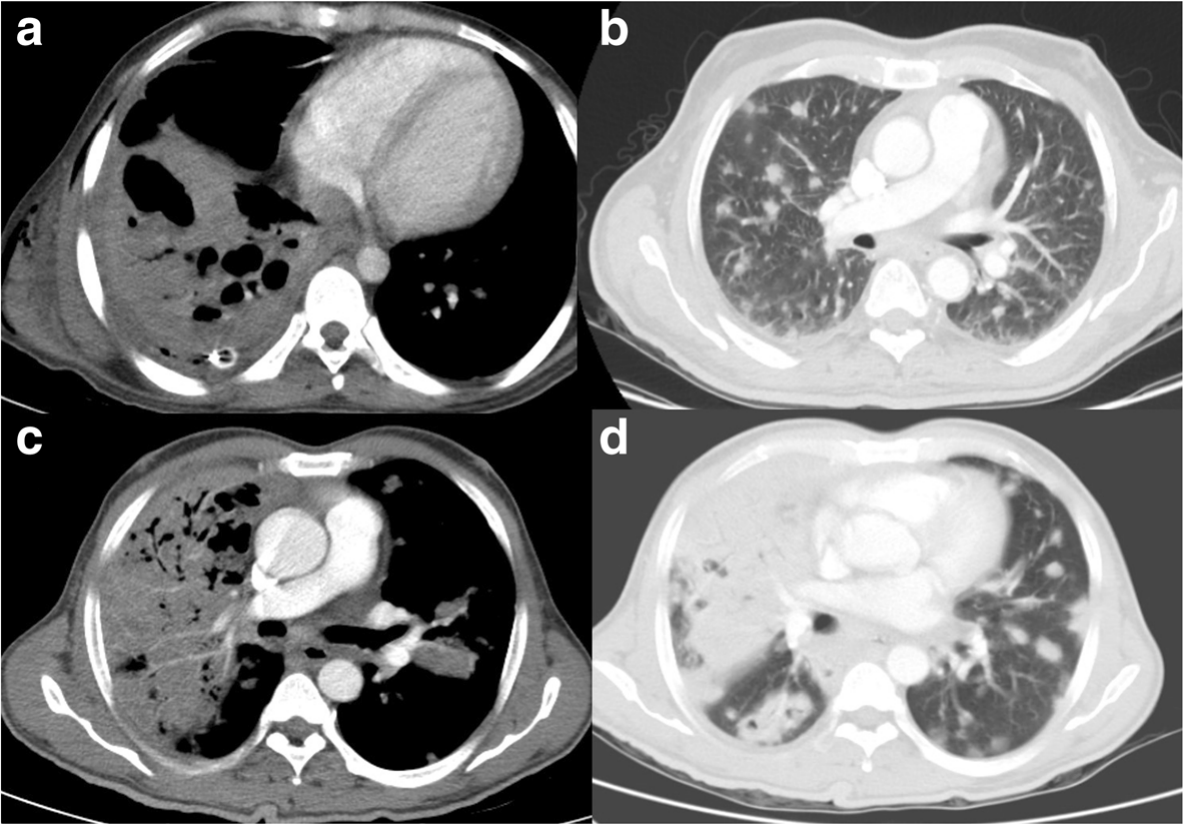
Lung melioidosis. **a** Forty-three-year-old male with a 1-month history of fever, respiratory symptoms, (**a**) axial CT thorax showed large consolidation with cavitation, intercostal drainage tube with residual mild right sided effusion, small chest wall collection. **b** Sixty-five-year-old male, known diabetic, presented with fever and low oxygen saturation at the time of admission, axial CT performed depicts multiple nodules in the lungs. This patient succumbed to death on day 6 of admission. Axial CT performed for a 49-year-old male presented with a 7-day history of fever, sputum culture grew Burkholderia pseudomallei, blood culture grew *Pseudomonas aeruginosa*, CT thorax in (**c**) mediastinal and (**d**) lung windows show large, multisegmental consolidation with cavitations and multiple nodules. Despite starting treatment for both organisms and being on ventilator support, he died on day 5 of admission

**Fig. 2:**
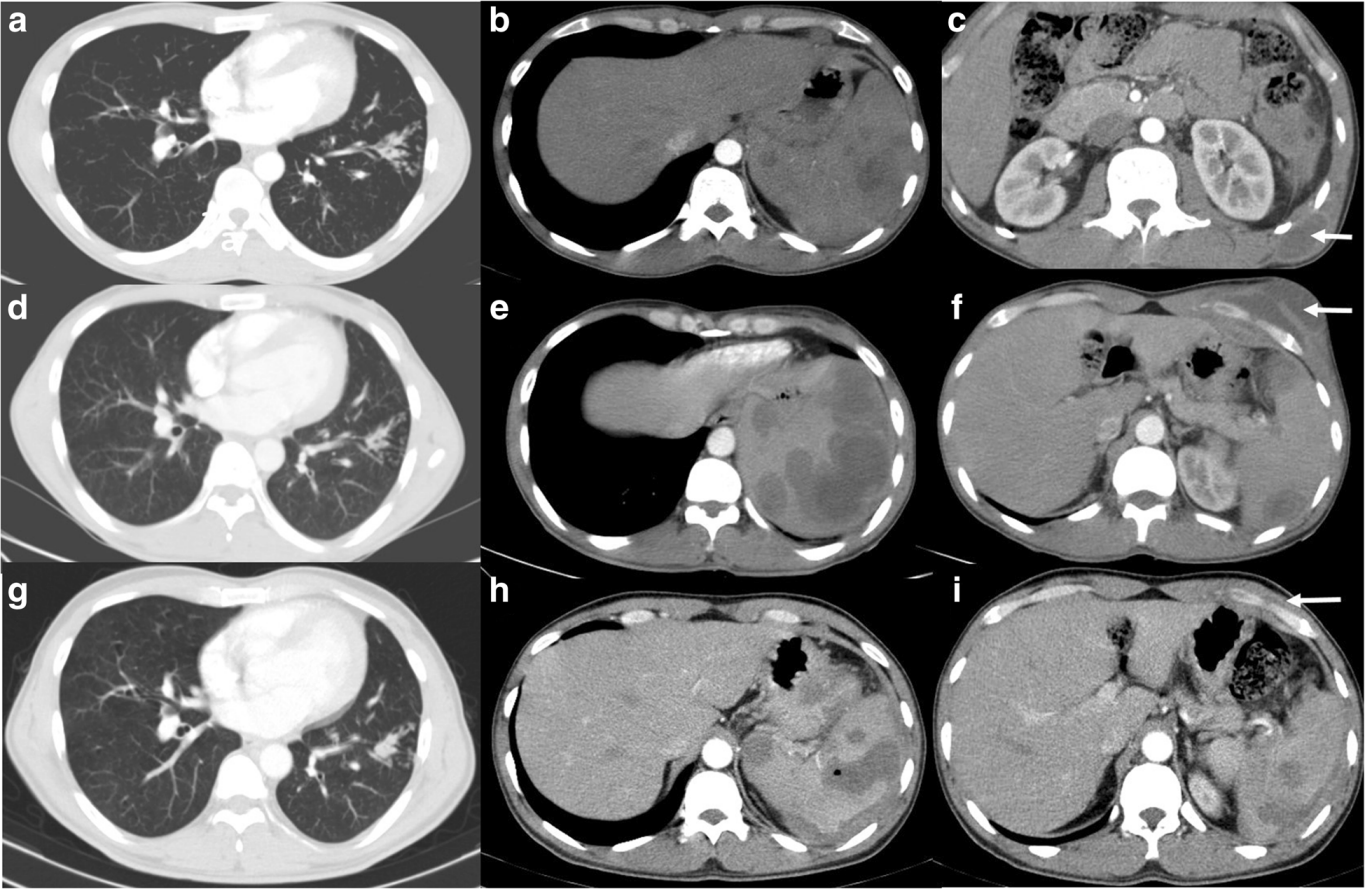
Chronic melioidosis with TB. Forty-one-year-old gentleman, newly diagnosed to have diabetes mellitus, presented with respiratory symptoms, (**a**) CT thorax showed the tree in bud opacities in the left lower lobe. Upper abdomen sections reveal (**b**) multiple hypodense foci in the spleen suggestive of small abscesses and (**c**) a small abscess in the left posterior lower chest wall (arrow). Cytology from the chest wall showed necrotising granulomatous inflammation, and antituberculous treatment was started. Two years later, he presented with new onset of left anterior chest wall swelling, for which CT thorax and abdomen were performed which showed (**d**) mild reduction in the ‘tree in bud’ opacities, but (**e**) increase in the size and number of splenic abscesses and (**f**) interval development of the left anterior lower chest wall abscess (arrow). Pus culture from the chest wall grew *Burkholderia pseudomallei*. Follow-up CT after 6 months showed, (**g**) stable tree in bud opacities in the lung, (**h**) near complete resolution of the chest wall abscess (arrow) but with the presence of perisplenic fluid with persisting splenic lesions (**h** and **i**), suggestive of contained rupture

The common features of lung and pleural melioidosis were nodules, consolidations, lung abscess, pleural effusion and empyema (Figs. 1and 2). Most of the patients had combined lung and pleural findings. Consolidations were the most common finding in lung involvement, followed by nodules. Cavitation within consolidation was noted in 7 cases. Pleural effusion was commonly associated with consolidation or lung nodules; however, five patients had pleural effusion without any lung findings.

### Abdomen

**Fig. 3:**
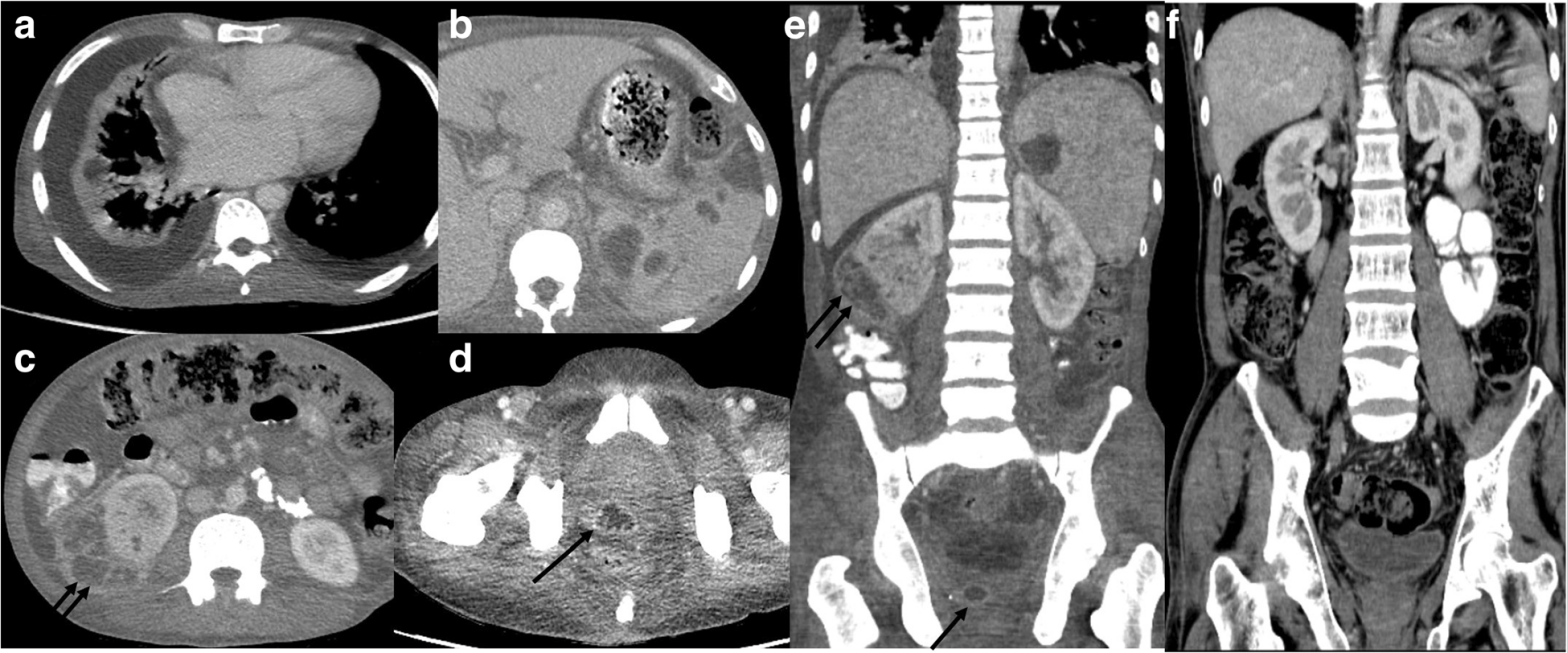
Disseminated melioidosis. Forty-three-year-old gentleman with pyrexia of unknown origin and anaemia, axial CT lower thorax reveal (**a**) bilateral pleural effusion with small evolving abscess in the atelectatic lung, bilateral pleural effusion, (**b**) multiple splenic abscesses, (**c**) large right renal abscesses with large subcapsular collection (double arrow), (**d**) prostatic abscess (single arrow). **e** Coronal CT abdomen images also demonstrate the same findings. **f** Follow-up CT of the abdomen after 6 months revealed complete resolution of the abscesses in various organs

In the abdomen, the spleen is the most common solid visceral organ involved, followed by the liver. The solid organ abscesses were seen as discrete and mostly multiple, single in a few cases. Most of the splenic abscesses were multiple, small in size and discrete, and a few were large and demonstrated multiple loculations within the abscess cavity as well as features of rupture resulting in localised peritoneal inflammation (Figs. 2 and 3). Contiguous chest or abdominal abscess was noted in eight subjects. Abscess in the liver was seen as part of disseminated disease except in one case, where the liver abscess was the sole imaging abnormality. The larger liver abscesses were observed to have multiple septations within, giving a ‘honeycomb’ appearance. Subcapsular location of visceral abscess predisposed to rupture and localised peritoneal reaction. Abscesses and inflammatory changes in close proximity to the portal venous system causing thrombosis in the veins were also noted, particularly in the presence of associated pancreatitis.

In this cohort, genitourinary system involvement was noted only in disseminated melioidosis (Fig. 3). The abscesses were seen in the kidneys, prostate and seminal vesicles. Renal abscesses were seen mostly as non-enhancing hypodense lesions, though one patient had large, multiloculated appearance, similar to splenic and liver abscesses. Prostatic abscess in one case was seen to contiguously involve the seminal vesicles. Associated cystitis was seen in one case. Prostatic abscesses were small and multiloculated.

### Head and neck

CNS manifestations were seen as meningitis, extra-axial fluid collection and brain abscess in dissemination from other sites of primary infection and as contiguous involvement with skull base osteomyelitis incited by overlying soft tissue or ear infection. Regional nodal involvement was seen in 10 to 15% of abdominal and thoracic manifestations.

### Bones and soft tissue

Isolated soft tissue involvement were demonstrated as cellulitis and abscess. Contiguous soft tissue involvement secondary to underlying bone involvement were also seen. The knee joint was the most commonly involved location for septic arthritis. The femur was most commonly involved with osteomyelitis. Amongst the six patients with psoas abscesses, three had spondylodiscitis of adjacent lumbar vertebrae.

Unusual sites of infection seen in our study included abscess in the pharynx, tonsils, submandibular nodes, mycotic aneurysm in the abdominal aorta, adrenal gland and seminal vesicle involvement as contiguous from adjacent organ involvement.

Table 1 is a summary of the imaging manifestations in the study sample.

**Table 1:** Imaging manifestation of various organ/system involvement (mutually inclusive)

Organ/system involved	Manifestation (*n*, %)
Lungs and pleura (*n* = 74)	Consolidation (19, 9.8) Nodules (10, 5.2) Consolidation and nodules (7, 3.6) Pleural effusion (8, 4.1) Combination of the lung and pleural findings (30, 15.5)
Spleen (*n* = 89)	Splenomegaly (24, 12.4) Splenic abscess (57, 29.4) Splenic abscess and perisplenic fluid/abscess rupture (8, 4.1)
Liver (*n* = 55)	Hepatomegaly (32, 16.5) Liver abscess (23, 11.9)
Bones and soft tissue (*n* = 65)	Abdominal and chest wall collection (19, 9.8) Osteomyelitis (9, 4.6) Joint effusion/septic arthritis (16, 8.2) Multiple joints, bones and soft tissue involvement (8, 4.1) Other soft tissue involvement (13, 6.7)
Genitourinary tract (*n* = 27)	Pyelonephritis/cystitis (13, 6.7) Renal abscess (3, 1.5) Prostatic abscess (7, 3.6) Seminal vesicle involvement (1, 0.5) Both renal and prostatic abscesses (3, 1.5)
Pancreas (*n* = 6)	Pancreatitis (4, 2.1) Focal pancreatic abscess (2, 1)
Lymph nodes (*n* = 32)	Thoracic nodes (7, 3.6) Abdominal nodes (16, 8.2) Thoracic and abdominal nodes (5, 2.6) Cervical nodes (4, 2.12)
Intra-abdominal free fluid (*n* = 24)	Present (23, 11.9)
CNS (*n* = 11)	Meningitis alone (1, 0.5) Brain abscess +/− meningitis (4, 2.1) Contiguous involvement from adjacent structures (Otitis, skull bone osteomyelitis, etc.) (6, 3.1)
Others (*n* = 11)	Adrenal involvement (1, 0.5) Cardiovascular including venous thrombosis (9, 46) Pharyngeal involvement (1, 0.5)
No abnormality in the imaging studies	(7, 3.6)

#### Disease outcome

Twenty-one percent (39 patients) of the study population suffered unfavourable outcome; amongst which 8.5% (16 patients) had death from disease. Fatalities were due to septic shock (*n* = 10), respiratory failure (*n* = 2) and cardiac arrest (*n* = 4). Lung involvement is independently associated with 3.2 times risk of unfavourable outcome (95% CI 1.54–6.63) whereas lymph node involvement was found to significantly predict favourable outcome (Table 2). When assessed specifically for mortality, lung involvement showed significant association with death (OR = 2.84[95% CI 1.54–6.63]; *p* = 0.05) as compared to other organ involvement.

**Table 2:** Association of organ involvement with favourable/unfavourable outcome

Organ involvement	Outcome		Chi-square test	Logistic regression		
	Favourable, *n* (%)	Unfavourable, *n* (%)	*p* value	*ß*-coefficient	Odds ratio (95% CI)	*p* value
Lungs and pleura						
Absent	100 (87)	15 (13)	0.001	1.16	3.20 (1.54–6.63)	0.002
Present	50 (68)	24 (32)				
CNS						
Absent	142 (80)	36 (20)	0.70	0.39	1.48 (0.37–5.85)	0.58
Present	8 (73)	3 (27)				
Liver						
Absent	110 (82)	24 (18)	0.15	0.54	1.72 (0.82–3.60)	0.15
Present	40 (73)	15 (27)				
Spleen						
Absent	76 (76)	24 (24)	0.23	− 0.44	0.64 (0.31–1.32)	0.23
Present	74 (83)	15 (17)				
Pancreas						
Absent	146 (80)	37 (20)	0.61	0.68	1.97 (0.35–11.18)	0.44
Present	4 (67)	2 (33)				
Gut						
Absent	129 (80)	33 (20)	0.83	0.11	1.12 (0.42–2.99)	0.83
Present	21(78)	6(22)				
Musculoskeletal system						
Absent	96 (77)	28 (23)	0.36	− 0.35	0.69 (0.32–1.51)	0.36
Present	54 (83)	11 (17)				
Lymph nodes						
Absent	120 (76)	37 (24)	0.02	− 1.53	0.22 (0.05–0.95)	0.04
Present	30 (94)	2 (6)				
TB						
Absent	130 (78)	37 (22)	0.26	− 1.05	0.35 (0.08–1.57)	0.17
Present	20 (91)	2 (9)				

OR (95% CI), odds ratio (95% confidence interval)

There was no significant association with clinical outcome in patients with disseminated disease or acute disease in our study population. No statistically significant association was found between the presence of diabetes as a risk factor and unfavourable outcome or mortality. Patient with diabetes mellitus was more vulnerable to the spleen and musculoskeletal involvement (Table 3).

**Table 3:** Association of organ involvement in melioidosis with diabetes mellitus

Organ involved	Diabetes mellitus		Chi-square test	Logistic regression		
	Absent, *n* (%)	Present, *n* (%)	*p* value	*ß*-coefficient	Odds ratio (95% CI)	*p* value
Lungs and pleura						
Absent	39 (34)	76 (66)	0.58	− 0.17	0.84 (0.46–1.55)	0.58
Present	28 (38)	46 (62)				
CNS						
Absent	61 (34)	117 (66)	0.20	− 0.83	0.43 (0.13–1.48)	0.18
Present	6 (55)	5 (45)				
Liver						
Absent	52 (39)	82 (61)	0.13	0.53	1.69 (0.85–3.36)	0.13
Present	15 (27)	40 (73)				
Spleen						
Absent	47 (47)	53 (53)	0.000	*1.12*	3.05 (1.62–5.77)	0.001
Present	20 (23)	69 (78)				
Pancreas						
Absent	65 (35)	118 (65)	1.0	0.09	1.10 (0.19–6.17)	0.91
Present	2 (33)	4 (67)				
Gut						
Absent	60 (37)	102 (63)	0.29	0.52	1.68 (0.67–4.21)	0.27
Present	7 (26)	20 (74)				
Musculoskeletal system						
Absent	51 (41)	73 (59)	0.02	*0.76*	2.14 (1.09–4.17)	0.03
Present	16 (25)	49 (75)				
Lymph nodes						
Absent	56 (36)	101 (64)	0.89	0.06	1.06 (0.47–2.35)	0.89
Present	11 (34)	21 (66)				

Association between TB and melioidosis was analysed by grouping as positive and negative TB based on the presence or absence of past history of tuberculosis or concurrent tuberculosis. Interestingly, none in the positive TB group had genitourinary tract involvement. There was significantly lesser number of patients with liver involvement in the positive TB group, which was statistically significant (*p* = 0.05).

## Discussion

Early and accurate diagnosis of melioidosis is warranted due to high fatality of melioidosis which could be as high as 80% amongst those with ineffective antibiotics and up to 50 to 90% amongst septic melioidosis [16, 25]. Hence, imaging is a complementary diagnostic tool as well as a predictor of disease outcome and mortality to guide patient management. This study, being one of the largest cohorts with culture-proven melioidosis, described the various imaging manifestations of melioidosis and prognosticate the clinical outcome. There is limited published literature which has evaluated the association of imaging features of meloioidosis with clinical outcome.

Multiple abscesses are the most frequent imaging manifestation of melioidosis [18]. The presence of respiratory system involvement showed significant association with a more severe form of infection with unfavourable outcome and predicted higher risk of mortality independently, compared to other organ or system involvement. Involvement of the lungs was most prevalent in our study population, as in previous studies [26–28]. Contradictorily, pleural effusion, not a previously described major feature, was seen in more than half of our cases. Perhaps this could be because smaller effusions are being more discernible on cross-sectional studies as compared to plain radiography. While a significant number of pleural effusions were part of the constellation of lung findings, a small number were reactive to splenic or liver manifestations of the disease.

Patients with diabetes mellitus were statistically more prone for infection of the bones and soft tissue. This explains the most common involvement of the bones and soft tissue in our cohort who had a 75% prevalence of diabetes as a risk factor. The proportion of the bones and joint involvement in our study population and the commoner affectation of the lower limbs to upper limbs agree with previous observations by other researchers [29, 30]. Additionally, infection of the bones and soft tissues was found to have statistically significant association with no mortality in our study population. Imaging pattern of appendicular or axial bone involvement was non-specific from other causes of infection. Soft tissue involvement was seen as abscesses with surface breakdown resulting in a site for discharging pus.

There was also significant association between diabetic mellitus and splenic abscess. The spleen was the most common organ to manifest abscess foci in disseminated disease; no splenic involvement was seen in isolation. Other studies have also made this observation with note that splenic lesions occurring along with an infective focus in the body would favour the diagnosis of melioidosis [19, 31, 32] especially in a diabetic patient. It is possible that disseminated disease is common amongst patients with diabetes mellitus where the infection spreads readily to the spleen.

Solid organ abscesses with internal septations giving the abscess a honeycomb appearance have been described in previous studies as well [18, 20, 33, 34]; while these observations were made in the liver, similar characteristics were seen in renal and prostate abscesses as well. Pancreatic involvement is seen as focal pancreatitis or contiguous involvement in disseminated disease with adjacent thrombophlebitis as has been described in earlier studies [35]. The reason for the absence of genitourinary tract involvement in disseminated melioidosis, in those patients with past history or concurrent tuberculosis, could not be ascertained in this study. Neurological manifestations were seen in 5% of our study population, the proportion is similar to previous studies in Northern Australia [36]. When regional lymph nodes were involved, it predicted overall favourable outcome which was statistically significant.

Tuberculosis is a close mimicker of melioidosis on imaging. Other pyogenic infections also share some of the imaging characteristics as melioidosis. The lung nodules in melioidosis are commonly larger and irregular. Lung nodules in active tuberculosis are commonly smaller, miliary nodules being typical. In our cohort, we did not encounter isolated lung cavities, though there were cavities within the consolidation. Melioid cavities are thin walled, and air-fluid levels are unusual findings, as compared to tuberculosis [37]. Isolated pleural effusion or pleural calcification could be seen with tuberculosis as well as melioidosis but uncommon in other pyogenic infection. Melioid abscesses in the liver could be larger or smaller. Honeycomb sign is found to be specific for melioidosis [38]. Abscesses in the liver and spleen are not commonly encountered in tuberculosis, whereas they are common with other pyogenic infections. Larger pyogenic abscesses can also demonstrate honeycomb sign, though not specific. Superficial soft tissue abscess along with deep solid visceral organ abscess is common with melioidosis and pyogenic infection, but uncommon with tuberculosis. Superficial soft tissue abscess along with underlying bone or joint infection is common with melioidosis and tuberculosis but uncommon with other pyogenic infections.

The radiologist has the potential to be the first to alert the clinician and microbiologist for enhanced diagnosis. Being a retrospective study, the timeline of clinical presentation and imaging investigation may not be uniform in our cohort of patients. Hence, any difference in the imaging manifestation of various organ involvement with respect to the duration of illness cannot be evaluated in our study. The pattern of resolution of imaging findings could not be determined in most of the cases due to the absence of follow-up imaging.

## Conclusion

In this large cohort of culture-proven melioidosis, we have described the spectrum of imaging manifestation of melioidosis and evaluated the association between clinical outcome and organ involvement as evaluated on imaging. Respiratory system involvement in melioidosis showed significant association with unfavourable outcome and predicted higher risk of mortality independently. Honeycomb sign which is specific for melioid liver abscesses is found in other solid organ melioid abscesses. Diabetes mellitus being a common risk factor for melioidosis is more prone for infection of the spleen and musculoskeletal system. Thus, awareness of imaging manifestations of melioidosis can complement microbiological diagnostic tests for accurate early diagnosis and timely medical management of melioidosis. Hence, imaging is an essential tool to assess the extent of disease, prognosticate and influence overall patient management and clinical outcome.

## Abbreviations

CT: Computed tomography
ICU: Intensive care unit
MRI: Magnetic resonance imaging
SD: Standard deviation
